# Identification of novel motif patterns to decipher the promoter architecture of co-expressed genes in *Arabidopsis thaliana*

**DOI:** 10.1186/1752-0509-7-S3-S10

**Published:** 2013-10-16

**Authors:** Yosvany López, Ashwini Patil, Kenta Nakai

**Affiliations:** 1 Department of Computational Biology, Graduate School of Frontier Sciences, The University of Tokyo, 5-1-5 Kashiwanoha, Kashiwa-shi, Chiba-ken 277-8561, Japan; 2 Human Genome Center, The Institute of Medical Science, The University of Tokyo, 4-6-1 Shirokane-dai, Minato-ku, Tokyo 108-8639, Japan

## Abstract

**Background:**

The understanding of the mechanisms of transcriptional regulation remains a challenge for molecular biologists in the post-genome era. It is hypothesized that the regulatory regions of genes expressed in the same tissue or cell type share a similar structure. Though several studies have analyzed the promoters of genes expressed in specific metazoan tissues or cells, little research has been done in plants. Hence finding specific patterns of motifs to explain the promoter architecture of co-expressed genes in plants could shed light on their transcription mechanism.

**Results:**

We identified novel patterns of sets of motifs in promoters of genes co-expressed in four different plant structures (PSs) and in the entire plant in *Arabidopsis thaliana*. Sets of genes expressed in four PSs (flower, seed, root, shoot) and housekeeping genes expressed in the entire plant were taken from a database of co-expressed genes in *A. thaliana*. PS-specific motifs were predicted using three motif-discovery algorithms, 8 of which are novel, to the best of our knowledge. A support vector machine was trained using the average upstream distance of the identified motifs from the translation start site on both strands of binding sites. The correctly classified promoters per PS were used to construct specific patterns of sets of motifs to describe the promoter architecture of those co-expressed genes. The discovered PS-specific patterns were tested in the entire *A. thaliana *genome, correctly identifying 77.8%, 81.2%, 70.8% and 53.7% genes expressed in petal differentiation, synergid cells, root hair and trichome, as well as 88.4% housekeeping genes.

**Conclusions:**

We present five patterns of sets of motifs which describe the promoter architecture of co-expressed genes in five PSs with the ability to predict them from the entire *A. thaliana *genome. Based on these findings, we conclude that the positioning and orientation of transcription factor binding sites at specific distances from the translation start site is a reliable measure to differentiate promoters of genes expressed in different *A. thaliana *structures from background genomic promoters. Our method can be used to predict novel motifs and decipher a similar promoter architecture for genes co-expressed in *A. thaliana *under different conditions.

## Background

Transcription is one of the most important biological processes taking place in the cell. Its control is carried out by a set of proteins known as Transcription Factors (TFs), which can regulate the expression of genes in specific tissues through their binding to DNA regulatory elements in nearby genomic regions [1]. Therefore, the study of TFs and their transcription factor binding sites (TFBSs) has turned out to be a key factor in understanding the regulation of transcription. Much attention has recently been devoted not only to predict TFBSs but also to model the binding and function of TFs in different tissues [2]. Many studies have attempted to elucidate aspects such as the binding process, the promoter structure and its regulatory elements in different ways. Stormo *et al. *regarded DNA sequences as vertices of a regular simplex in order to explain the binding mechanism [3]; whereas Barash *et al. *employed Bayesian network representations of TFBSs to expand the probabilistic representation of DNA motifs from an independent position specific-scoring matrix to a full dependency model [4]. On the other hand, Carninci *et al. *sequenced tags of several TFBSs in mouse and human genomes to analyze the evolution of different promoter classes, thus identifying new transcription start sites (TSSs) that facilitated the identification of tissue-specific promoters and their *cis-*acting elements [5]. Smith *et al. *studied proximal promoters of human and mouse genes across differentiated tissues to identify regulatory modules capable of differentiating changes in expression and thus explain tissue-specific differential expression [6]. Other works have focused specifically on *cis-*regulatory modules (CRMs), even though unknown functional solitary sites could be ruled out. Sharov *et al. *identified potential CRMs defined as groups of conserved TFBSs in the entire mouse genome [7]. Li *et al. *found common properties that might help in the identification of CRMs and the understanding of their function. They reported that CRMs do indeed share common features such as elevated GC contents, increased levels of interspecific sequence conservation, and tendency to be transcribed into RNA [8]. Loo *et al. *proposed an algorithm for detecting CRMs in groups of co-expressed genes. Their predictions showed a high enrichment of CRMs close to the TSS for differentiated tissues versus a depletion of them for embryonic development gene sets in this region [9]. Segal *et al. *designed a thermodynamic model for computing expression patterns where *cis-*regulatory sequences, binding-site preferences and expression of TFs were taken into account. Their model, validated in *Drosophila melanogaster*, accurately predicted expression patterns of CRMs and showed the presence of positional information in regulatory sequences [10]. Since promoters might contain a variety of TFBSs for different TFs, it is no longer enough to think of these entities acting individually. Regarding the dependency among TFBSs and the hypothesis that genes showing similar expression profiles could share common structural characteristics in their regulatory regions, Vandenbon *et al. *proposed a simple Markov chain-based promoter architecture model as an alternative to the CRM approaches. Their method included characteristics such as orientation, position with respect to the translation start site (TLS) and order of predicted occurrences of over-represented motifs [11]. However the motif patterns of promoter regions in plants have been inadequately analyzed. Since the analysis of promoter regions is also easier in genomes with short intergenic regions, we chose the *Arabidopsis thaliana *genome to conduct our analysis. Previously, Molina and Grotewold made use of a combination of expectation-maximization and Gibbs sampling methods to identify motifs over-represented in *A. thaliana *core promoters [12]. However they did not focus on the combination of their predicted motifs to identify patterns of sets of motifs within promoters of co-expressed genes in specific structures of this organism.

In this study, we used the distance and orientation of motifs over-represented in four different plant structures (PSs) and in the whole *A. thaliana *to build specific motif patterns in order to capture the promoter region of co-expressed genes. We predicted motifs specific to four different PSs and to the entire *A. thaliana *where 8 of them did not match significantly to *cis-*acting regulatory elements stored in the PLACE database [13] and were thus considered novel motifs. In the next step, five novel patterns of motif combinations that describe the promoter architecture of genes expressed in "flower", "seed", "root", "shoot" and the "whole plant" were built. Each pattern identified a significant number of genes expressed in petal differentiation, synergid cells, root hair and trichome; as well as housekeeping genes from the whole *A. thaliana *genome. These results indicate the presence of patterns and the suitability of our approach to identify them.

## Results

We used here the ATTED-II, which is a database of co-expressed *A. thaliana *genes deduced from microarray data [14]. After getting specific groups of co-expressed genes, each was split into two further subsets: a set to predict motifs and another to choose the best PS-specific promoters and build novel motif patterns (see Methods section). In order to find a similar promoter architecture for co-expressed genes in four different *A. thaliana *structures and the whole plant, we started our analysis by predicting motifs (see Methods section for detailed description) with key regulatory roles in the following PSs: flower, seed, root, shoot and the entire plant.

### Selection of PS-specific motifs

**Figure 1:**
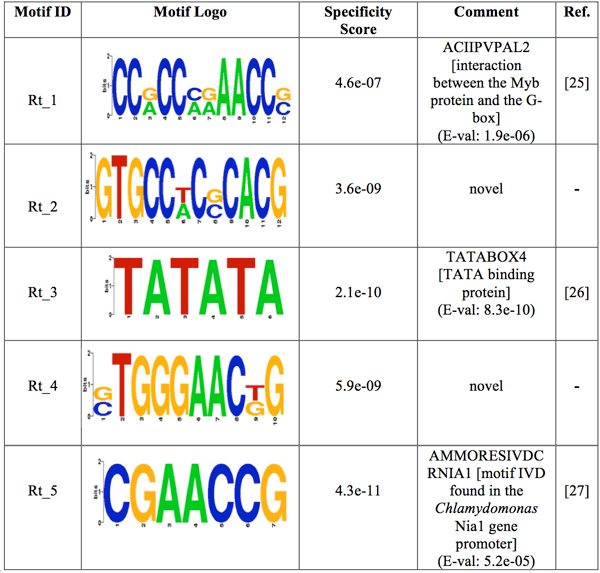
**Logos of the over-represented motifs in root**. For each motif, its group specificity score and a comment is included. A known motif is also depicted with an E-val from the STAMP website application [24], a description of the TF binding to it and its reference.

**Figure 2:**
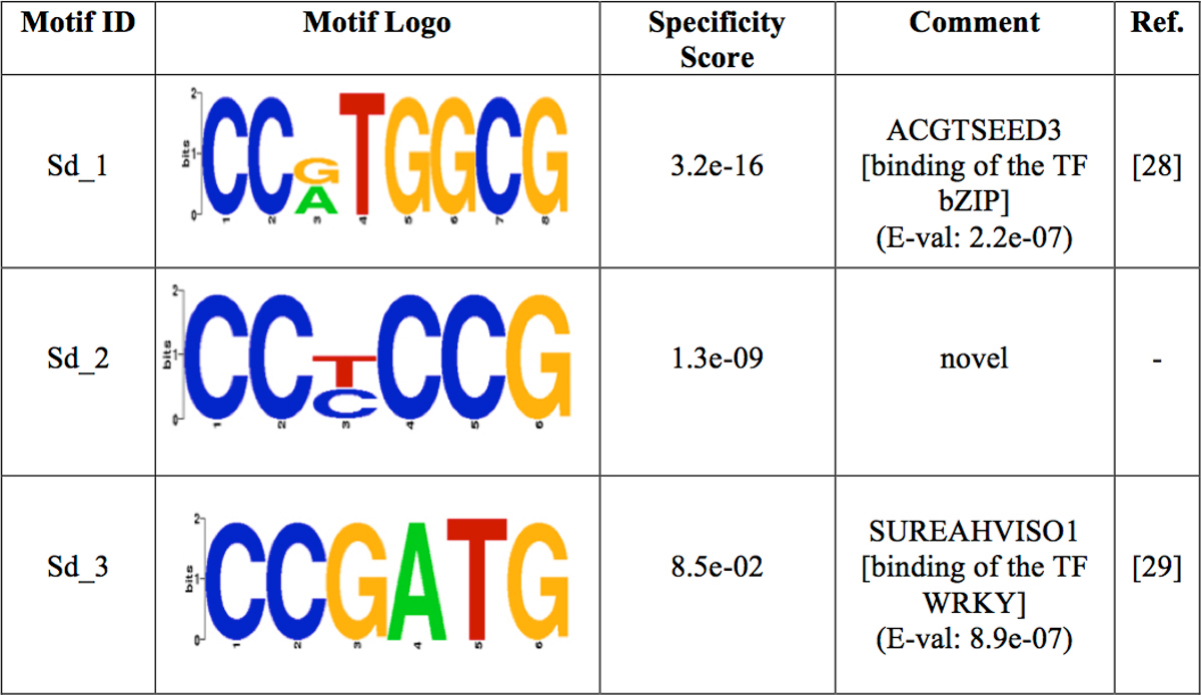
**Logos of the over-represented motifs in seed**. For each motif, its group specificity score and a comment is included. A known motif is also depicted with an E-val from the STAMP website application [24], a description of the TF binding to it and its reference.

**Figure 3:**
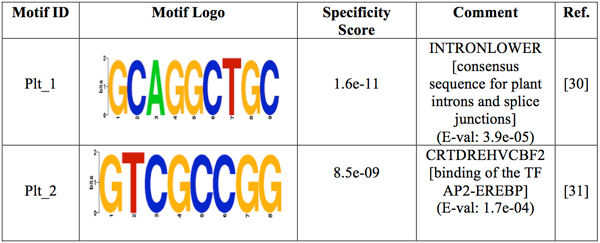
**Logos of the over-represented motifs in whole plant**. For each motif, its group specificity score and a comment is included. A known motif is also depicted with an E-val from the STAMP website application [24], a description of the TF binding to it and its reference.

**Figure 4:**
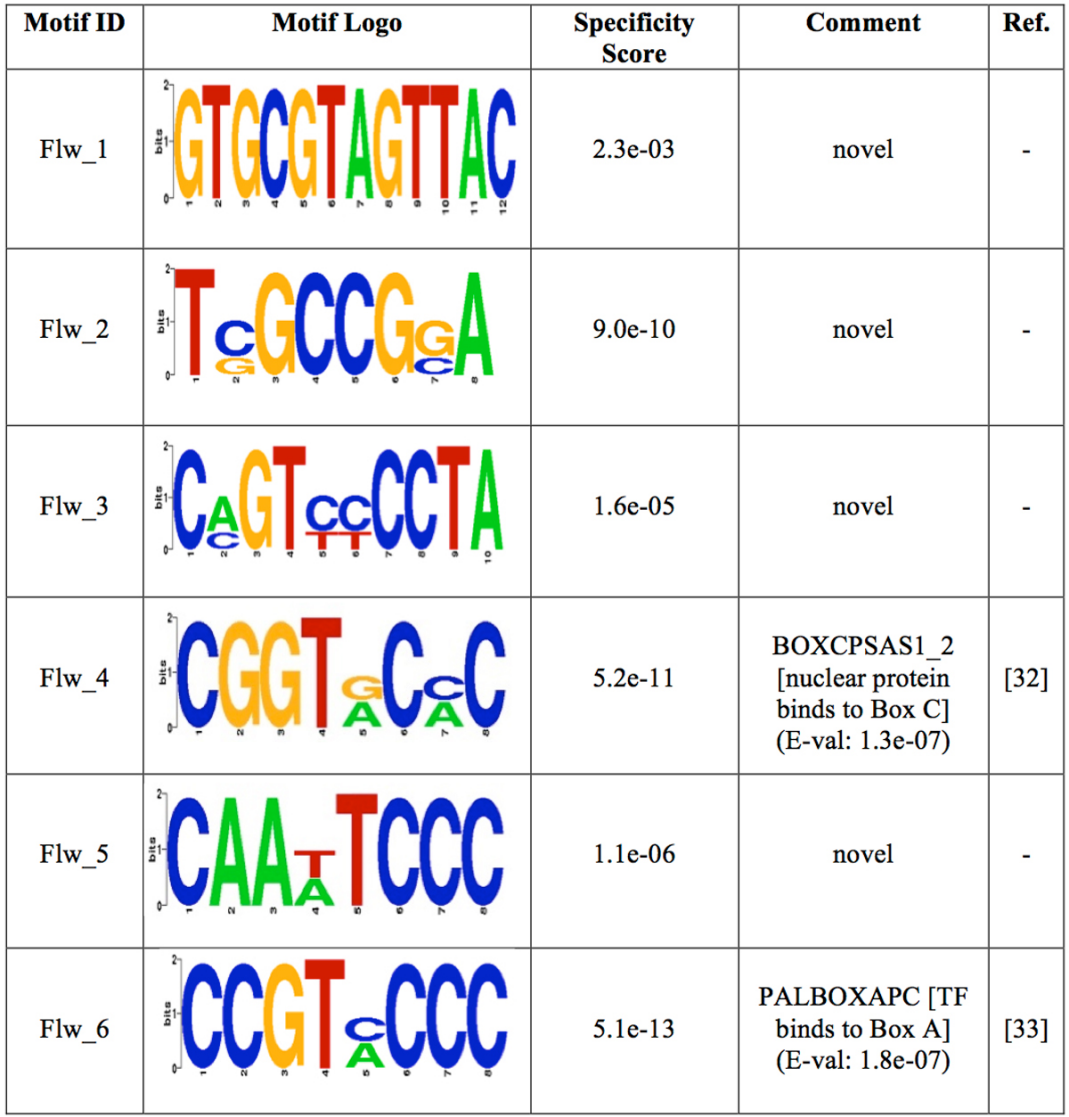
**Logos of the over-represented motifs in flower**. For each motif, its group specificity score and a comment is included. A known motif is also depicted with an E-val from the STAMP website application [24], a description of the TF binding to it and its reference.

**Figure 5:**
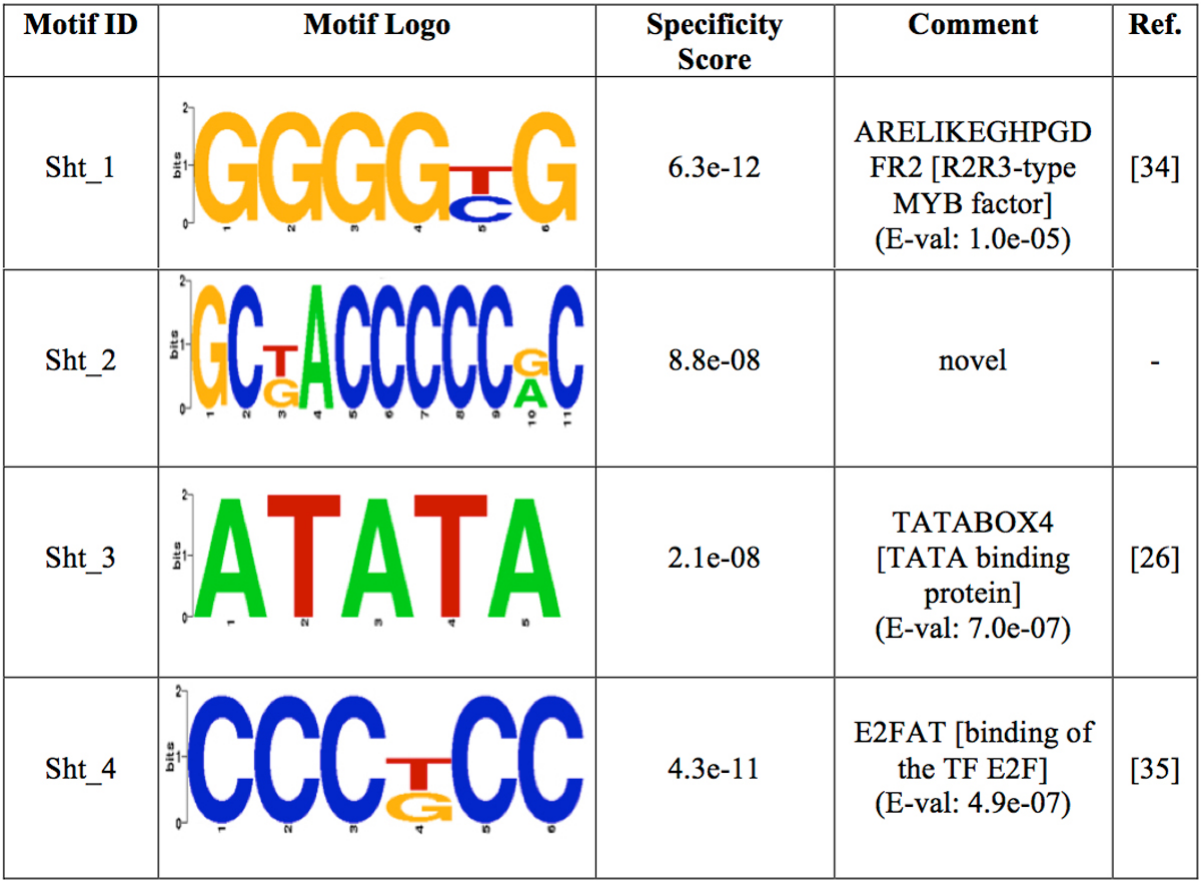
**Logos of the over-represented motifs in shoot**. For each motif, its group specificity score and a comment is included. A known motif is also depicted with an E-val from the STAMP website application [24], a description of the TF binding to it and its reference.

The motif-prediction process per set of promoters identified 142 flower-specific motifs, 183 seed-specific motifs, 171 root-specific motifs, 142 shoot-specific motifs and 141 whole plant-specific motifs, respectively (see table 1). To remove redundant motifs, each position frequency matrix was converted to a *k-*mer frequency vector that was then used to build a distance matrix by the Pearson Correlation distance. This matrix was used to cluster each group of PS-specific motifs by average-linkage hierarchical method. The optimal number of clusters per PS was 6, 3, 5, 4 and 2 for flower, seed, root, shoot and whole plant, respectively (see table 1). Hereafter the whole plant will be referred to as a PS for simplicity. The group specificity score (measure of how well a motif targets the promoter regions where it was found) [15] of each motif was computed and motifs with the smallest score per cluster were chosen for further analysis. The selected motifs were further compared with plant *cis-*acting regulatory elements in the PLACE database [13]. Motifs with *p-*values less than 0.001 were regarded as known motifs, otherwise, novel ones. In order to restrict as much as possible our motif comparison, we chose a strict *p-*value equal to that successfully used to validate the motif comparison algorithm TOMTOM (see additional data file in [16]). As a result, motif Rt_1 (see Figure 1) matched to ACIIPVPAL2 (motif known for playing a key role in vascular tissue whose primary component "xylem" is usually located close to the interior of roots), motif Sd_1 (see Figure 2) matched to ACGTSEED3 ("ACGT motif" related to seed expression) and motif Plt_1 (see Figure 3) matched to INTRONLOWER (motif involved in "3' intron-exon splice junctions" in the plant). On the contrary, flower-specific motifs Flw_1, Flw_2, Flw_3 and Flw_5 (see Figure 4), root-specific motifs Rt_2 and Rt_4 (see Figure 1), seed-specific motif Sd_2 (see Figure 2) and shoot-specific motif Sht_2 (see Figure 5) did not match significantly to any known *cis-*acting regulatory element in the PLACE database, thus representing potentially new regulatory motifs in plants. We also compared our predicted motifs with others previously reported in *A. thaliana *[12]. As a result, motif Plt_2 (see Figure 3) matched to Motif_8 (see Figure 1 in [12]), motif Rt_3 (see Figure 1) matched to Motif_3 (see Figure 1 in [12]) and motif Sd_1 (see Figure 2) matched to Motif_11 (see Figure 1 in [12]) with *p-*values less than 0.001. In addition, we compared our 8 novel motifs to those stored in JASPAR database [17] and found that all the compared plant motifs matched significantly to motifs in other organisms (see table 2).

**Table 1:** Detailed information of each PS-specific model

Models	Gene Sets		Motif Predictions		Accuracy^♣^ (%)	Genes predicted genome-wide^♠^
			
	motif-prediction^†^	model-build^‡^	overall*	over-represented^§^		
**Flower**	55	83	142	6	75.8	**49/**63
**Seed**	59	88	183	3	69.0	**134/**165
**Root**	64	95	171	5	65.2	**34/**48
**Shoot**	62	92	142	4	60.2	**51/**95
**Whole Plant**	58	87	141	2	64.1	**76/**86

Notes

**^† ^**and **^‡ ^**columns indicate the number of genes in the "motif-prediction" and "model-build" sets

^* ^column shows the number of motifs predicted by the three motif-discovery programs

**^§ ^**column indicates the amount of over-represented motifs per model

**^♣ ^**column shows the accuracy achieved by the corresponding SVMs

**^♠ ^**column depicts the amount of genes predicted genome-wide

**Table 2:** Information regarding the comparison with motifs of other organisms. For each novel plant motif, the TF of the motif it matched to with an E-val from the STAMP website application [24], the organism the TF was found in and a reference is shown

Novel plant motifs	Comment	Organism	**Ref**.
Flw_1	ladybird early homeodomain TF (lbe) (E-val: 2.40e-06)	*D. melanogaster*	[36]

Flw_2	regulatory protein CAT8 (E-val: 5.95e-05)	*S. cerevisiae*	[37]

Flw_3	probable transcription repressor RGM1 (E-val: 3.54e-05)	*S. cerevisiae*	[38]

Flw_5	TF c-Rel (E-val: 3.36e-07)	*H. sapiens*	[39]

Sd_2	operator OpA (E-val: 5.39e-06)	*S. cerevisiae*	[40]

Rt_2	early growth response protein 1 (Egr1) (E-val: 9.86e-05)	*R. norvegicus*	[41]

Rt_4	suppressor of hairless homolog (Su_H) (E-val: 2.31e-05)	*C. intestinalis*	[42]

Sht_2	transcription corepressor MIG3 (E-val: 3.01e-06)	*S. cerevisiae*	[43]

### Classification of PS-specific promoters

Each group of training promoter regions was scanned for TFBSs of its PS-specific motifs on both strands and matrices (referred to as TRAINING MATRIX) composed of 12-component, 6-component, 10-component, 8-component and 4-component vectors characterizing the promoter regions in flower, seed, root, shoot and the whole plant were created. For each "TRAINING MATRIX", another matrix composed of background promoter regions, which were not included in both the "model-build set" and the "motif-prediction set" (see "Final promoter sets" in Methods section) of a PS was further formed. After training the support vector machine (SVM) with the corresponding matrices, each remaining single-promoter set was used for evaluating its performance (see table 1 and Additional File 1). Considering a leave one-out cross-validation approach, the SVM of the flower model achieved the highest accuracy of 75.8%. In addition, the SVMs of seed, root and plant models reached similar accuracies of 69.0%, 65.2% and 64.1%, whereas that of the shoot model achieved the lowest accuracy of 60.2%.

### Creation of novel motif patterns

Using the results of the SVM predictions, we next tried to create patterns composed of motif sets that may help us to decipher a similar architecture for promoters of co-expressed genes. For this, we used the promoter regions correctly classified by every SVM to create five distinct promoter sets in flower, seed, root, shoot and whole plant, respectively. Each previous promoter set was scanned for TFBSs of the respective PS-specific motifs within four different intervals: [0,-50], [-50,-100], [-100, -150] and [-150,-200] on both strands. The motifs present in more than 60% of promoters in flower, seed, root and shoot as well as 50% of promoters in whole plant were used to build patterns of sets of motifs to describe the promoters of co-expressed genes in the four PSs and the entire plant (see table 3).

**Table 3:** Novel patterns of sets of motifs in promoters of *A. thaliana*'s co-expressed genes

Promoter region	FLOWER			SEED			ROOT			SHOOT			PLANT		
	
	motif	+	-	motif	+	-	motif	+	-	motif	+	-	motif	+	-
0 to -50	Flw_3	*	*	Sd_1	∆	*	Rt_5	*	*	Sht_3	*	*	Plt_1	*	*
	Flw_4	*	*	Sd_2	*	*							Plt_2	∆	*
	Flw_5	*	*	Sd_3	∆	*									

-50 to -100	Flw_2	*	∆	Sd_1	∆	*	Rt_3	*	*	Sht_1	∆	*	Plt_1	*	*
	Flw_3	*	*	Sd_2	*	∆	Rt_4	∆	*	Sht_3	*	*	Plt_2	∆	*
	Flw_4	*	*	Sd_3	*	*	Rt_5	*	*	Sht_4	∆	*			
	Flw_5	*	*												

-100 to -150	Flw_2	*	*	Sd_2	*	*	Rt_3	*	*	Sht_1	∆	*	Plt_1	*	*
	Flw_3	*	∆	Sd_3	*	*	Rt_4	*	*	Sht_3	*	*	Plt_2	*	∆
	Flw_4	*	∆	Sd_1	*	∆	Rt_5	*	*	Sht_4	∆	*			
	Flw_5	*	*												

-150 to -200	Flw_3	*	∆	Sd_3	*	∆	Rt_1	*	∆	Sht_1	∆	*	Plt_1	*	*
	Flw_4	*	∆				Rt_5	*	*	Sht_3	*	*			
	Flw_5	*	*							Sht_4	∆	*			

Notes

"+" and "-" stand for the DNA strands

"*" and "∆" indicate presence and absence of the motif

### Flower-pattern

**Figure 6:**
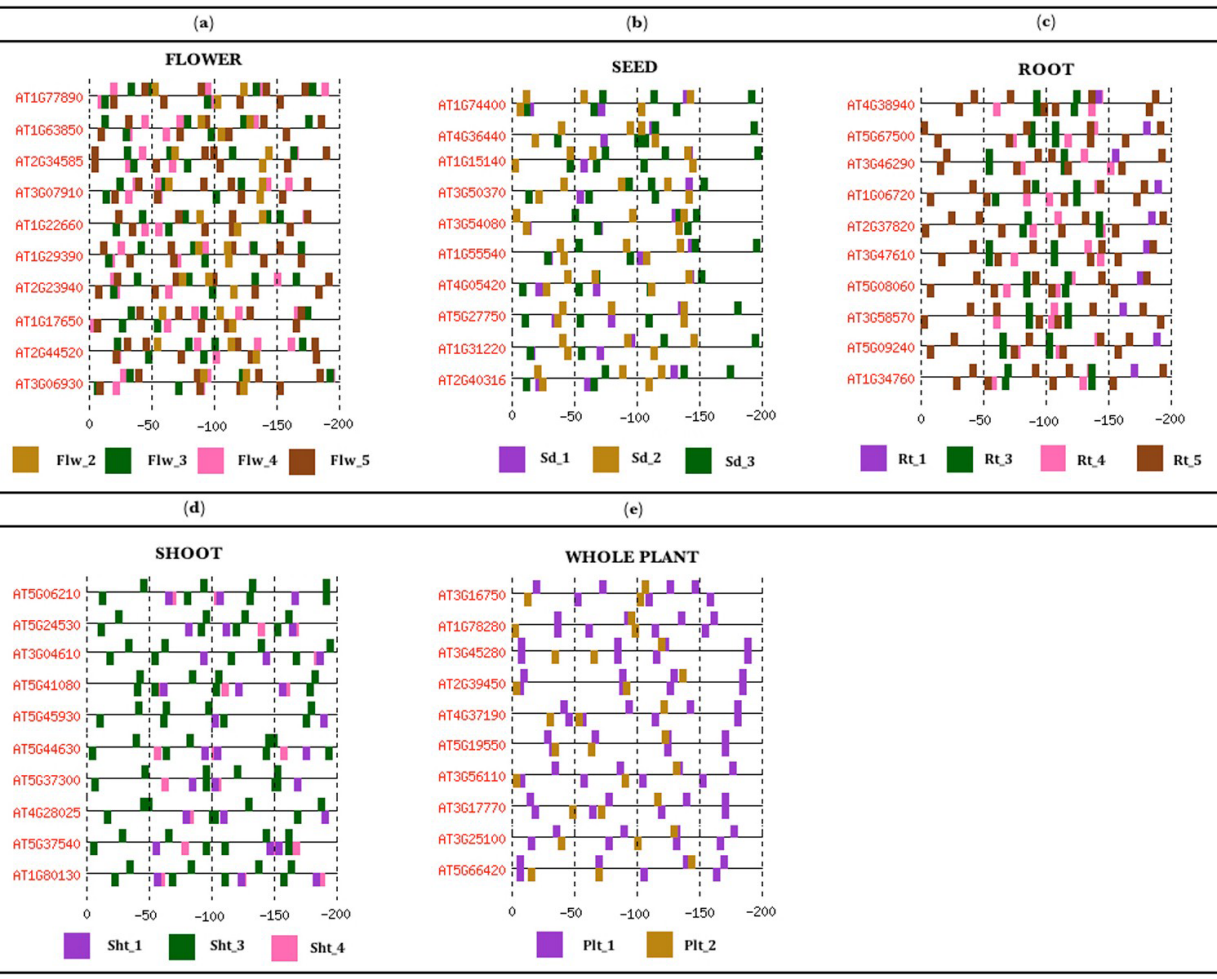
**Promoter architecture of uncovered genes expressed in four *A. thaliana *structures and the entire plant**. This figure shows the promoter of housekeeping genes and other genes expressed in four different *A. thaliana *structures. Such genes were identified by the patterns of sets of motifs proposed here. The promoter regions have been divided into four regions and those significant motifs within each region are shown (see Additional Files 2, 3, 4, 5, 6 for extended illustrations and a brief description of genes).

The pattern for promoters of genes expressed in flower comprises four motifs (see Figure 6a and Additional File 2). It was observed that motif Flw_5 has a strong tendency to be present throughout the promoter region on both strands, whereas motifs Flw_3 and Flw_4 have a tendency to be found on both strands at the region 0 to -100 near the translation start site (TLS). The presence of Flw_3 and Flw_4 at the core promoter region on both strands could possibly facilitate a stronger binding of the transcriptional machinery. On the other hand, motif Flw_2 has a tendency to be at the region -100 to -150 on both strands. Figure 6a (see Additional File 2) shows the promoter region of genes expressed in petal differentiation identified by our method. In addition, motifs Flw_1 and Flw_6 were present in less than 47% of promoters, so it is possible that their TFs do not act independently at specific distances from the TLS but their role in transcription might be related to the presence of other TFs with which they act in cooperation. Motif Flw_2 is, on the other hand, present on minus strand at the region 0 to -100 in 57.4% of promoters, whereas on both strands at the region -150 to -200 in 44.4% of promoters.

### Seed-pattern

The pattern for promoters of genes expressed in seed combines all the motifs over-represented in this PS (see Figure 6b and Additional File 3). Motif Sd_2 shows a tendency to appear on plus strand at the region -50 to -100, but on both strands at the region 0 to -50. The presence of motif Sd_3 is restricted to the region -50 to -100 on both strands, whereas motif Sd_1 tends to appear on minus strand at the region 0 to -100. Figure 6b (see Additional File 3) shows the promoter region of genes expressed in synergid cells. In addition, motif Sd_1 is sparsely present (less than 40% of promoters) on both strands at the region -150 to -200. Motif Sd_2, on the other hand, is also poorly represented (less than 35% of promoters) on both strands at the region -150 to -200.

### Root-pattern

The pattern for promoters of genes expressed in root combines the presence of four motifs (see Figure 6c and Additional File 4). Motif Rt_5 shows a strong tendency to be on both strands throughout the promoter region. Motifs Rt_3 and Rt_4 tend to appear at the region -100 to -150 on both strands and motif Rt_3 that significantly matched to TATABOX4 has a tendency to be bound at about the same distance reported for a TATA box. Figure 6c (see Additional File 4) shows the promoter region of genes expressed in root hair. Since motifs Rt_1 and Rt_2 are poorly present (less than 40% of promoters) at the region 0 to -50 on both strands, the TFs of both motifs might be somehow linked. The TF binding to motif Rt_5 seems to have an important role within the core promoter region, whereas the TFs of motifs Rt_3 and Rt_5 could be cooperating at specific distances from each other on both strands at the region -50 to -150.

### Shoot-pattern

The pattern for promoters of genes expressed in shoot combines the presence of three motifs (see Figure 6d and Additional File 5). Motif Sht_3 tends to appear throughout the promoter region on both strands, whereas motifs Sht_1 and Sht_4 show a tendency to be found at the region -50 to -200 on minus strand. Figure 6d (see Additional File 5) shows the promoter region of genes expressed in trichome. The fact that motifs Sht_1 and Sht_4 tend to be on the same strand at specific distances from the TLS may suggest not only a presence of their TFs at these specific positions but also at precise distances between them.

### Whole plant-pattern

The pattern for promoters of housekeeping genes expressed in the whole plant comprises the presence of only two motifs (see Figure 6e and Additional File 6). Motif Plt_1 tends to appear throughout the promoter region on both strands, whereas motif Plt_2 has a tendency to be found at the region 0 to -100 on minus strand. Figure 6e (see Additional File 6) shows the promoter region of plant housekeeping genes. Surprisingly, motif Plt_2 is poorly present (less than 8% of promoters) at the region -150 to -200 on plus strand, while its presence is more clearly visible near the core promoter region. It is possible that more than two TFs might be involved in the transcription of genes expressed in the whole plant, but our method of obtaining over-represented motifs might have ruled out their motifs.

### Genome-wide prediction of co-expressed genes

We used the above described motif patterns: "flower-pattern", "seed-pattern", "root-pattern", "shoot-pattern" and "whole plant-pattern" to search for genes expressed in each PS within the entire *A. thaliana *genome. We ruled out all the *A. thaliana*'s genes whose promoter regions were more than 60% similar. As a result, the initial set of 22 591 genes was reduced to 19 212 genes. We thus identified 63, 165, 48, 95 and 86 genes whose promoters satisfied the motif patterns. In order to illustrate the validity of our predictions we checked the plant ontology terms (POTs) per group of predicted genes. We found 49 (77.8%) out of 63 genes expressed in petal differentiation and expansion stage, 134 (81.2%) out of 165 genes expressed in synergid cells, 34 (70.8%) out of 48 genes expressed in root hair, 51 (53.7%) out of 95 genes expressed in trichome and 76 (88.4%) out of 86 genes with housekeeping function (see table 1). The poor accuracy of trichome could be due to similar promoter structures between genes expressed in "shoot" and those expressed in the other four PSs. Such similarity could impede the SVM from correctly differentiating the promoters of trichome-expressed genes.

## Discussion

From the 20 motifs predicted in promoters of genes expressed in the analyzed PSs, 8 of them did not match significantly to either *cis-*regulatory elements in the PLACE database [13] or previous plant motifs reported in [12]. This work reports novel patterns of sets of motifs capable of describing the promoter architecture of co-expressed genes in four distinct PSs and the entire plant *A. thaliana*. It regarded two features of promoter regions: orientation and distance of TFBSs from the TLS. Each PS-specific "motif-prediction set" was used to search for motifs. Those PS-specific over-represented motifs were employed to scan the promoter regions and compute specific features. Despite the lack of transparent results achieved by a SVM, its kernel allows flexibility in separating PS-specific promoters from background genomic promoters. Unlike artificial neural networks that give multiple solutions related to a local minimal and may not be robust enough over distinct instances, a SVM gives a unique solution considering the convexity of the optimization problem. Hence a SVM was trained to discriminate between PS-specific promoters and background genomic promoters. Those correctly classified promoters per PS were scanned for TFBSs of their over-represented motifs within four bins covering the entire promoter region: 0 to -50, -50 to -100, -100 to -150 and -150 to -200, thus defining five motif patterns: "flower-pattern", "seed-pattern", "root-pattern", "shoot-pattern" and "whole plant-pattern". Such patterns were used to scan the *A. thaliana *genome and uncovered 49, 134, 34 and 51 genes expressed in petal differentiation, synergid cells, root hair and trichome, and 76 housekeeping genes. Since TSS data is not available for *A. thaliana*, generally the distance between TSS and TLS is believed to be short in this species. A former study has also suggested the presence of more putatively functional motifs in the 5'UTR regions of *A. thaliana *than previously thought [18]. Our study encompasses two key points: (1) a support vector machine to discriminate promoters of genes expressed in four different PSs and in the whole plant from background genomic promoters and (2) novel patterns of sets of motifs able to successfully describe the promoter architecture of co-expressed genes in four PSs and in the entire *A. thaliana*.

## Conclusions

We have worked with promoter sets of genes expressed in four different *A. thaliana *structures and in the whole plant. Regulatory motifs specific to each promoter group were predicted and 8 of them with key regulatory functions in four PSs were potentially new and yet unknown motifs. In addition, five distinct patterns of sets of PS-specific motifs able to describe the promoter region of co-expressed genes were built and shown to be useful in predicting genes expressed in specific biological processes from the entire *A. thaliana *genome. To our knowledge, several works have attempted to elucidate the promoter architecture in different higher organisms, but none of them have been focused on plants. As the motif patterns indicate, the motifs along with their positioning and orientation within the TFBSs at specific distances from the TLS is a reliable measure to differentiate promoters of genes expressed in different *A. thaliana *structures from background genomic promoters. This method could be used to predict novel motifs and decipher a similar promoter architecture for genes co-expressed in *A. thaliana *in other tissues and conditions. We are trying to incorporate additional characteristics of promoters such as distance and order between motifs as to achieve promoter architecture models as broad as possible. Future analyses are expected to uncover novel regulatory motifs and a common promoter architecture for genes expressed in tissues or cells of different metazoans.

## Methods

**Figure 7:**
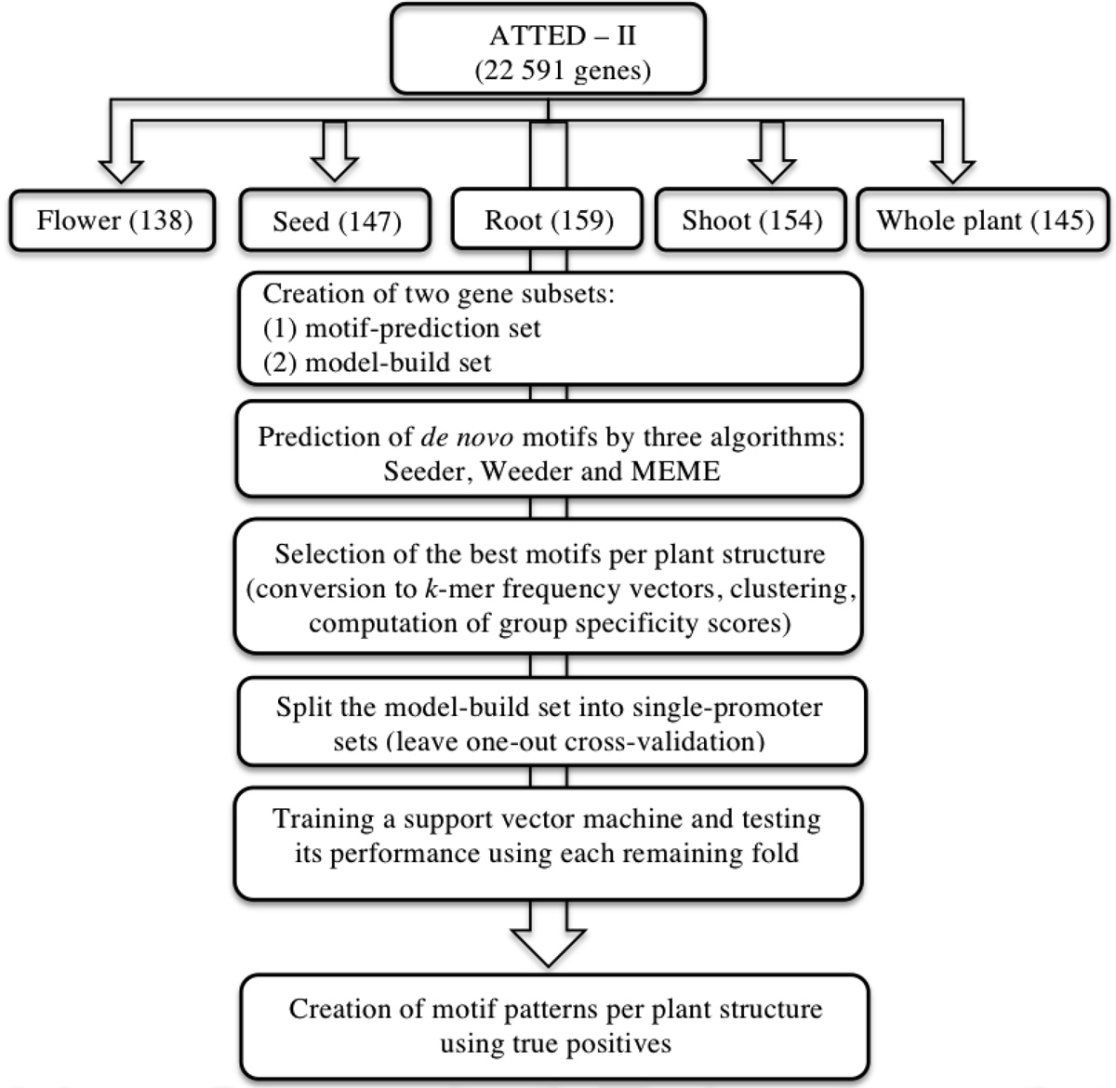
**Workflow of the used methodology**. This figure depicts the workflow of our methodology.

Figure 7 shows the workflow of our methodology.

### Initial database

We worked with the version TAIR10 (ftp://ftp.arabidopsis.org/home/tair/Sequences/whole_chromosomes) of the *A. thaliana *genome. In addition, an *A. thaliana trans*-factor and *cis-*element prediction database (ATTED-II) [14] comprising information of co-expressed genes deduced from microarray data was used. ATTED-II contains the expression of 22 591 genes in different experimental series.

Since we are interested in PS-specific genes, five distinct datasets composed of the normalized expression of 22 591 genes from 81, 27, 21, 27 and 9 microarrays based on annotation of flower, seed, root, shoot and whole plant were formed. Each gene set was further used to identify co-expressed genes and thus form reduced gene groups per plant structure (PS). The standard deviation of a gene's average expression values through all the PS-specific sets was computed. It was multiplied by a threshold (number manually chosen to get sets of over a hundred genes) and the resulting product was compared with the difference between the two greatest average expressions. As a result, the target gene is assigned to the PS-specific set in which its average expression was greatest. Thus, sets of 138, 147, 159, 154 and 145 genes expressed in flower, seed, root, shoot and in the whole plant were obtained with thresholds of 2.05, 2.35, 2.36, 0.80 and 0.75, respectively.

### Final promoter sets

Each reduced set composed of genes co-expressed in flower, seed, root, shoot and whole plant was split into two distinct subsets: (1) model-build set and (2) motif-prediction set which are randomly composed of 60% and 40% of genes in the corresponding original set. The model-build set was used to differentiate promoters containing a precise combination of motifs from background genomic promoters and thus build novel patterns of sets of motifs based on the correctly classified PS-specific promoters. The motif-prediction set was, on the other hand, employed to search for *de novo *motifs. The promoter regions stretching 50bp, 100bp, 150bp and 200bp upstream from the translation start site (TLS) [18] were considered and promoters of genes in the motif-prediction set were grouped using these distances. As a result, four different sets composed of promoters 50bp, 100bp, 150bp and 200bp long were created. For each PS-specific promoter set, an additional one (non-PS specific) composed of background genomic promoters other than those of genes in the subsets: motif-prediction set and model-build set was formed.

### Identification and comparison of motifs

We used three different motif-discovery programs: Seeder [19], Weeder [20] and MEME [21]. For Seeder [19], motifs 6bp, 8bp, 10bp and 12bp long with a seed length of 7 were predicted. Several runs were done regarding both strands. Weeder [20] was also run on both strands and the following motif lengths: 6bp with 1 mutation, 8bp with 2 and 3 mutations, 10bp with 3 and 4 mutations and 12bp with 4 mutations were searched. For MEME [21], motifs whose length was between 6bp and 12bp, and with any number of repetitions on both strands, were predicted. In order to remove redundant motifs, the position frequency matrix of each motif was converted into a *k-*mer frequency vector [22]. A distance matrix was then built by using the Pearson Correlation distance and PS-specific motifs were clustered according to their similarity by average-linkage hierarchical method. The optimal number of clusters was 6, 3, 5, 4 and 2. Additionally, each motif's group specificity score (measure of how well a motif targets the promoter regions where it was found) [15] per set of predicted motifs in flower, seed, root, shoot and whole plant was computed and the motif with the smallest score of every cluster was considered for further analysis. The chosen motifs were compared with plant *cis-*acting regulatory elements stored in the PLACE database [13] and motifs matching with *p-*values higher than 0.001 were regarded novel motifs.

### Characterization of promoter regions

**Figure 8:**
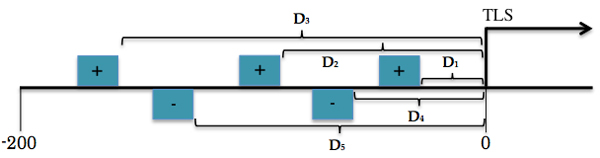
**Distribution of TFBSs for one specific motif along a promoter region**. This figure shows the hypothetical distribution of TFBSs for the same motif. Boxes with "+" are TFBSs located on plus strand, whereas those with "-" are positioned on minus strand. The D_*i*_'s stand for the distances of the TFBSs from the TLS.

The promoter of each gene in the model-build set was scanned to identify TFBSs for its PS-specific motifs. For every promoter, the average of the distances from the TLS per motif on both strands (see Figure 8) was computed as follows,

(1) $$AVG=\frac{\sum x}{n}$$

where *x *represents the distance from the TLS of a TFBS and *n *stands for the number of TFBSs on the same strand.

The promoter regions were characterized by different-size vectors depending on how many motifs are over-represented in the model under analysis. For instance, 6 motifs were chosen in "flower" (6 average distances in each strand for 6 motifs) hence the promoters of genes co-expressed in "flower" will be characterized by a 12-component vector. Furthermore, the distances were divided by the promoter's length (200bp) for normalization and the average distance of an absent motif on a specific strand was regarded to be zero. As a result, a matrix referred to as "TRAINING MATRIX" that characterizes the training promoter regions was prepared per promoter set.

### Training of a support vector machine

A support vector machine (SVM) [23] is a supervised-learning algorithm able to predict the class of a new instance (unknown category) once a set of objects that belong to two possible categories is given. This algorithm seeks a hyperplane that optimally separates instances of either class with a maximal distance (margen) from them. Each model-build set composed of PS-specific promoters was randomly split into single-promoter groups. By following the leave one-out cross-validation technique, each single-promoter set was employed for testing the model's performance, whereas the remaining groups were used for training the SVM. Accordingly, the number of feature vectors in the "TRAINING MATRIX" varies depending on how many promoters are used for training. The SVM's performance was thus evaluated using each remaining single-promoter set. Moreover, a testing promoter region is also characterized as previously explained in the section "Characterization of promoter regions".

### Creation of motif patterns using true positives

To decipher a similar promoter architecture for genes co-expressed in each PS and in the whole plant, we picked out the promoters (labeled as true positives) of genes expressed in each specific PS because they seem to share a similar architecture. The incorrectly classified promoters, on the other hand, do not seem to contain an alike architecture of interest to us. We thus attained five separate groups composed of 56, 55, 70, 60 and 63 promoters of genes expressed in flower, seed, root, shoot and whole plant, respectively. We regarded four distinct regions: [0, -50], [-50, -100], [-100, -150] and [-150, -200] which cover the entire promoter region and calculated each PS-specific motif's distribution through both strands per promoter group. Those motifs present in more than 60% of promoters in flower, seed, root and shoot sets as well as 50% of promoters in whole plant set were regarded for further analysis. As a result, we formed five specific patterns of sets of motifs to decipher the promoter architecture of those co-expressed genes.

### Genome-wide prediction of genes co-expressed in four PSs and the entire plant

The promoter regions with more than 60% of similarity were removed from the initial set of 22 591 genes, thus resulting a final group of 19 212 genes. Each identified motif pattern was used to predict genes expressed within one of the four PSs and the entire plant. Although genes whose promoters were employed to train each model were not ruled out, no overlapping was detected after the genome-wide prediction was carried out. Predictions were tested for accuracy using their plant ontology annotations for cellular location.

## List of abbreviations used

TLS: Translation Start Site; TFs: Transcription Factors; TFBSs: Transcription Factor Binding Sites; CRMs: *Cis*-regulatory modules; SVM: Support Vector Machine; PS: Plant Structure; POTs: Plant Ontology Terms.

## Competing interests

The authors declare that they have no competing interests.

## Authors' contributions

YL conceived and developed the methodology, designed the SVM, and drafted the manuscript under the guidance of AP, and KN supervised the whole research. All authors read and approved the final manuscript.

## Supplementary Material

Additional file 1 **Information of each SVM's performance**. Click here for file

Additional File 2 **Promoter region of 27 out of 49 genes involved in petal differentiation found with the "flower-pattern"**. Click here for file

Additional File 3 **Promoter region of 29 out of 134 genes expressed in synergid cells found with the "seed-pattern"**. Click here for file

Additional File 4 **Promoter region of 29 out of 34 genes expressed in root hair found with the "root-pattern"**. Click here for file

Additional File 5 **Promoter region of 29 out of 51 genes expressed in trichome found with the "shoot-pattern"**. Click here for file

Additional File 6 **Promoter region of 29 out of 76 housekeeping genes found with the "whole plant-pattern"**. Click here for file

## Declarations

Publication of this article is supported by the Cabinet Office, Government of Japan and the Japan Society for the Promotion of Science (JSPS) through the Funding Program for World-Leading Innovative R&D on Science and Technology (FIRST Program).

This article has been published as part of *BMC Systems Biology *Volume 7 Supplement 3, 2013: Twelfth International Conference on Bioinformatics (InCoB2013): Systems Biology. The full contents of the supplement are available online at http://www.biomedcentral.com/bmcsystbiol/supplements/7/S3.
