# Supplementary material for: Identification of novel motif patterns to decipher the promoter architecture of co-expressed genes in Arabidopsis thaliana
Source: BMC Syst Biol. 2013 Oct 16;7(Suppl 3):S10. doi: 10.1186/1752-0509-7-S3-S10 (PMC3852273; doi:10.1186/1752-0509-7-S3-S10)
Supplement: Additional file 1 — Information of each SVM's performance. [file 1752-0509-7-S3-S10-S1.pdf]

Results of each support vector machine

Model	TP	TN	FN	FP	Sensitivity	Specificity	Accuracy (%)
**Flower**	56	63	27	11	0.675	0.851	75.8
**Seed**	55	63	33	20	0.625	0.759	69.0
**Root**	70	46	25	37	0.737	0.554	65.2
**Shoot**	60	46	32	38	0.652	0.548	60.2
**Whole Plant**	63	44	24	36	0.724	0.55	64.1

Notes
TP: True Positives
TN: True Negatives
FN: False Negatives
FP: False Positives
