# Supplementary material for: Identification of novel motif patterns to decipher the promoter architecture of co-expressed genes in Arabidopsis thaliana
Source: BMC Syst Biol. 2013 Oct 16;7(Suppl 3):S10. doi: 10.1186/1752-0509-7-S3-S10 (PMC3852273; doi:10.1186/1752-0509-7-S3-S10)

Promoter region of 27 out of 49 genes involved in petal differentiation, which were found with the "flower-pattern". Such regions illustrate the positioning of motifs: Flw_2, Flw_3, Flw_4 and Flw_5 on both strands at specific distances from the translation start site. For each gene, a brief description of its function is also provided

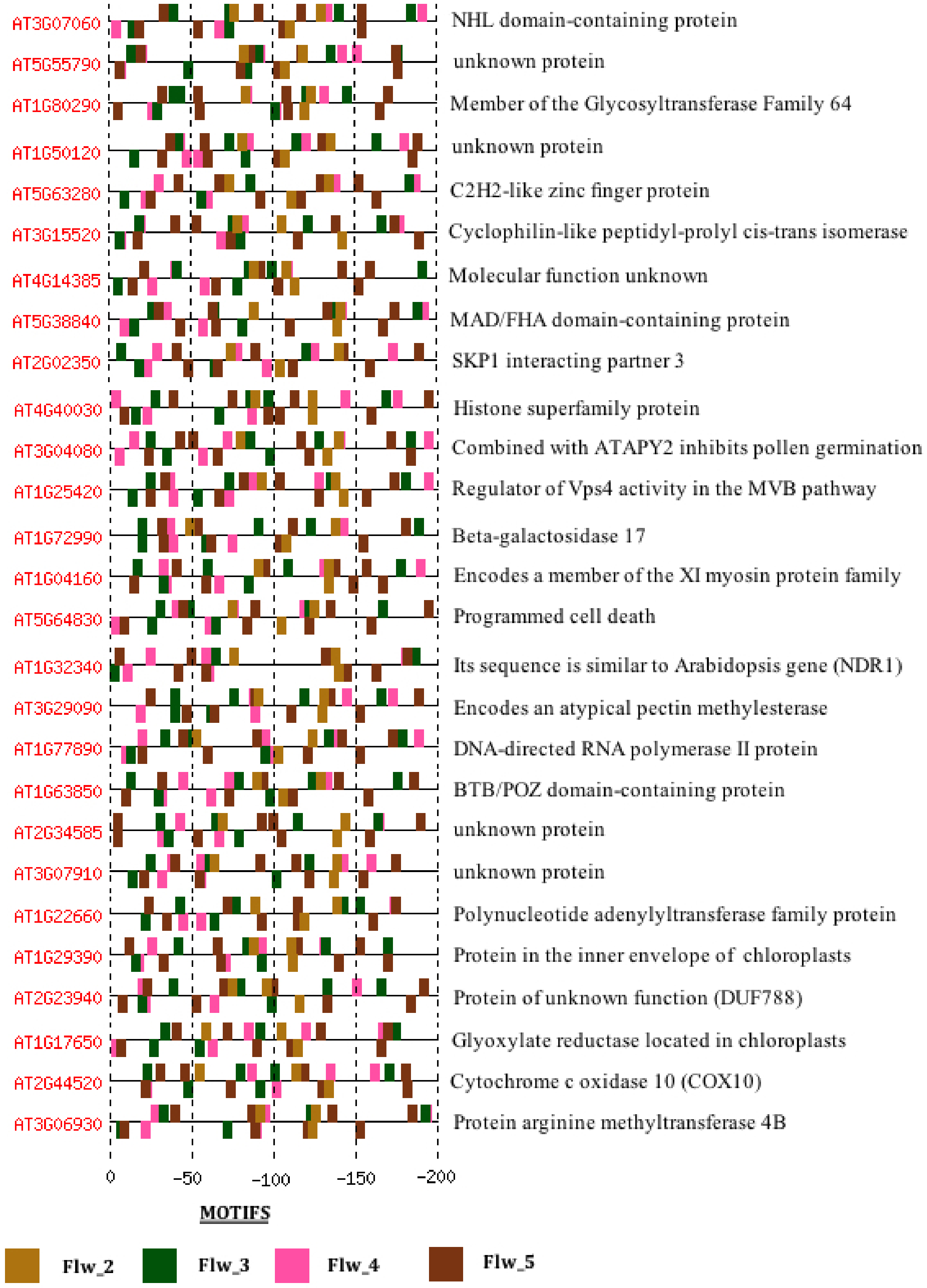

Supplement: Additional File 2 — Promoter region of 27 out of 49 genes involved in petal differentiation found with the "flower-pattern". [file 1752-0509-7-S3-S10-S2.pdf]
