# Supplementary material for: Identification of novel motif patterns to decipher the promoter architecture of co-expressed genes in Arabidopsis thaliana
Source: BMC Syst Biol. 2013 Oct 16;7(Suppl 3):S10. doi: 10.1186/1752-0509-7-S3-S10 (PMC3852273; doi:10.1186/1752-0509-7-S3-S10)

Promoter region of 29 out of 34 genes expressed in root hair, which were found with the "root-pattern". Such regions illustrate the positioning of motifs: Rt_1, Rt_3, Rt_4 and Rt_5 on both strands at specific distances from the translation start site. For each gene, a brief description of its function is also provided

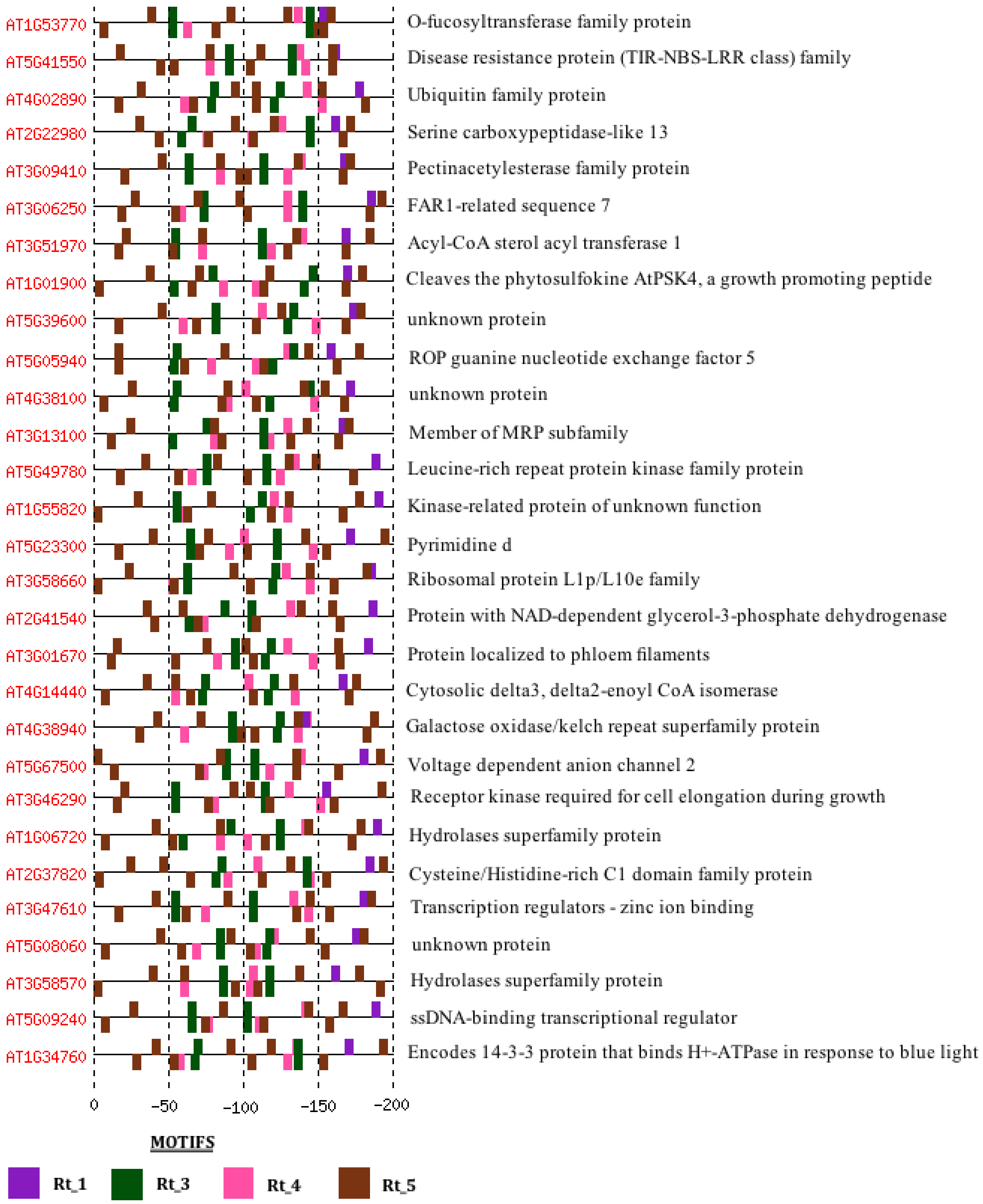

Supplement: Additional File 4 — Promoter region of 29 out of 34 genes expressed in root hair found with the "root-pattern". [file 1752-0509-7-S3-S10-S4.pdf]
